# Strong and Specific Recognition of CAG/CTG Repeat DNA (5’‐dWGCWGCW‐3’) by a Cyclic Pyrrole‐Imidazole Polyamide

**DOI:** 10.1002/cbic.202100533

**Published:** 2021-11-18

**Authors:** Yuki Hirose, Tomo Ohno, Sefan Asamitsu, Kaori Hashiya, Toshikazu Bando, Hiroshi Sugiyama

**Affiliations:** ^1^ Department of Chemistry Graduate School of Science Kyoto University Kitashirakawa-oiwakecho Sakyo-ku Kyoto 606-8502 Japan; ^2^ Present address: Department of Genomic Neurology Institute of Molecular Embryology and Genetics Kumamoto University, Honjo Chuo-ku Kumamoto 860-0811 Japan; ^3^ Institute for Integrated Cell-Material Science (iCeMS) Kyoto University, Yoshida-ushinomiyacho Sakyo-ku, countryPart/>Kyoto 606-8501 Japan

**Keywords:** CAG/CTG repeats, DNA recognition, pyrrole-imidazole polyamides, triplet repeats

## Abstract

Abnormally expanded CAG/CTG repeat DNA sequences lead to a variety of neurological diseases, such as Huntington's disease. Here, we synthesized a cyclic pyrrole‐imidazole polyamide (cPIP), which can bind to the minor groove of the CAG/CTG DNA sequence. The double‐stranded DNA melting temperature (*T*_m_) and surface plasmon resonance assays revealed the high binding affinity of the cPIP. In addition, next‐generation sequencing showed that the cPIP had high specificity for its target DNA sequence.

## Graphical abstract

**A cyclic pyrrole‐imidazole** polyamide, which binds to the CAG/CTG repeat sequence with high specificity and affinity, is reported.

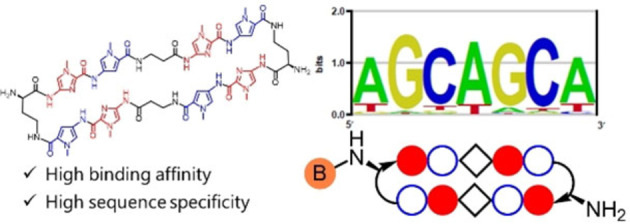

Although DNA repeat sequences are normally present in the human genome, abnormally elongated repeats can lead to a variety of diseases.[^1^, ^2^] The abnormal elongation of CAG/CTG repeat sequences causes Huntington's disease, spinocerebellar ataxia, and myotonic dystrophy. Compounds that bind to the CAG/CTG repeat sequences have been studied to develop therapeutic agents for these neurological disorders.[^3^, ^4^, ^5^] Notably, Pearson et al. recently achieved a reduction in the number of repeats *in vivo* using a compound that binds to the hairpin structure formed by the CAG repeats.^[6]^ These studies targeted the r(CUG) repeats[^3^, ^4^] or d(CAG/CAG) hairpin structures in the CAG repeat regions,[^5^, ^6^] whereas our group previously developed pyrrole‐imidazole polyamides (PIPs) that bind to the d(CAG/CTG) sequences in a sequence‐specific manner.[^7^, ^8^]

PIPs are one of the well‐studied DNA‐binding compounds, which were developed by Dervan et al.[^9^, ^10^] They bind to the minor groove of B‐DNA, recognizing Watson‐Crick base pairs by antiparallel pairings of their *N*‐methylpyrrole (Py) and *N*‐methylimidazole (Im) moieties: a Py/Im pair recognizes a C/G pair and a Py/Py pair recognizes an A/T or T/A pair. A γ‐aminobutyric acid turn (γ‐turn) connects two arrangements of Py and Im to create hairpin PIPs (hPIPs), which have been frequently used in many studies. For effective binding of long (more than 10 heteroaromatic rings) hPIPs, a β‐alanine moiety is used as a substitute for a Py to ease the curvature of the hPIPs: a Py/β and β/β pair recognizes an A/T or T/A pair.^[11]^ Addition of a second γ‐turn to close the hairpin structure results in cyclic PIPs (cPIPs). In previous studies, cPIPs with eight rings were reported to have higher DNA‐binding affinity than the corresponding hPIPs.[^12^, ^13^] In terms of the specificity of base recognition, an 8‐ring cPIP distinguished a single base mismatch better than an hPIP.^[12]^

To create a ligand that binds to the CAG/CTG repeat with high affinity and specificity, we designed and synthesized cPIP **1**, which targets the sequence 5′‐WGCWGCW‐3′. We also synthesized hPIP **2** with the same recognition sequence for comparison (Figure 1). The (*R*)‐α‐substituted γ‐turn was used to enhance the binding affinity and orientation specificity of each PIP.[^14^, ^15^] The synthetic routes of PIPs **1** and **2** are shown in the Experimental Section.

**Figure 1:**
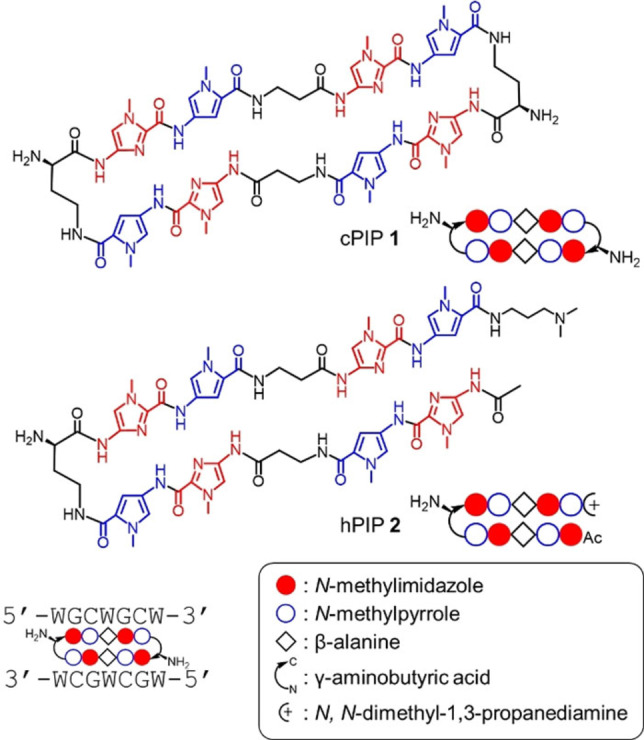
Chemical structures and ball‐and‐stick notation of cPIP **1** and hPIP **2**.

First, we evaluated the binding affinity of PIPs **1** and **2** for their target sequence using the double‐stranded DNA melting temperature (*T*_m_) assay with a sequence 5′‐CG**AGCAGCA**CG‐3′/3′‐GC**TCGTCGT**GC‐5′ (bases in bold font are recognized by the PIPs). The measured *T*_m_ and Δ*T*_m_ (Δ*T*_m_=*T*_m_[DNA+PIP]−*T*_m_[DNA]) values are presented in Table 1 and the representative melting curves are in Figure S5. As expected, cPIP **1** showed a higher binding affinity than hPIP **2** (ΔΔ*T*_m_=12.4 °C). To further investigate the binding property of these PIPs, we performed *T*_m_ assays using 5′‐(CAG)_10_‐3′ (5′‐CAGCAGCAGCAGCAGCAGCA‐GCAGCAGCAG‐3′) and 5′‐(CTG)_10_‐3′ (5′‐CTGCTGCTGCTGCT‐GCTGCTGCTGCTGCTG‐3′) sequences and obtained values that are shown in Table 1 and Figure S6. Each of these sequences forms a self‐complementary hairpin structure, which contains A/A or T/T mismatch pairs. Expanded CAG/CTG repeat sequences are known to partially form such structures.^[16]^ Interestingly, although there are three A/A or T/T mismatches per binding site, both of these PIPs bound to 5′‐(CAG)_10_‐3′ and 5′‐(CTG)_10_‐3′ sequences strongly. As with the CAG/CTG sequence, cPIP **1** also showed a higher binding affinity than hPIP **2** in this case (ΔΔ*T*_m_=9.4 °C and 6.9 °C, respectively). These findings indicated that cPIP **1** could bind to CAG/CTG repeat sequences even when the sequences formed unnatural structures. Moreover, findings of the *T*_m_ assays using 5′‐r(CAG)_10_‐3′ and 5′‐r(CUG)_10_‐3′ clearly indicated that these PIPs bound with high specificity to DNA rather than RNA (Figure S7).

**Table 1:** *T*_m_ and Δ*T*_m_ values of compounds **1** and **2**.

	Match		5’‐(CAG)_10_‐3’		5’‐(CTG)_10_‐3’	
	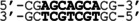		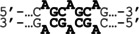		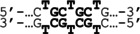	
PIP	*T*_m_/°C	Δ*T*_m_/°C	*T*_m_/°C	Δ*T*_m_/°C	*T*_m_/°C	Δ*T*_m_/°C
–	41.9 (±0.5)	–	49.0 (±0.7)	–	50.6 (±0.3)	–
cPIP **1**	93.2 (±0.2)	51.2 (±0.6)	92.7 (±0.5)	43.7 (±0.8)	92.0 (±0.6)	41.4 (±0.7)
hPIP **2**	80.7 (±0.4)	38.8 (±0.7)	83.2 (±0.6)	34.3 (±0.9)	85.1 (±0.4)	34.5 (±0.5)

In addition, we performed the surface plasmon resonance (SPR) assay to obtain the kinetic constants for the interaction between these PIPs and their target DNA. A 5′‐biotin‐labeled hairpin DNA (5′‐biotin‐CGCG**AGCAGCA**CGCGTTTTCGCG**TGCTGCT**CGCG‐3′) was immobilized on a streptavidin‐coated sensor chip, and various concentrations of the polyamide solution were added (Figure S8). The sensorgrams shown in Figure 2 were obtained using the single‐cycle program on the Biacore T200 system. The rates of association (*k*_a_) and dissociation (*k*_d_) and the dissociation constant (*K*_D_) are listed in Table 2 and other detailed values in Figure S9. Notably, the U‐values, which indicate the reliability of the data, were large in each case. This might be due to the linearity of the association and dissociation regions, the large R_max_, and the *k*_d_ values below the specification range of the system (Figure S9). Surprisingly, contrary to the findings of our *T*_m_ assay, cPIP **1** showed a larger *K*_D_ value than hPIP **2**. We do not have a certain explanation for this inconsistency, but it might be due to differences in the measurement methods and PIP concentrations. Interestingly, these two PIPs showed different kinetic properties: cPIP **1** exhibited lower *k*_a_ and *k*_d_ values than hPIP **2**. Therefore, although hPIP **2** binds to the target DNA faster than cPIP **1**, it is more difficult to dissociate the latter than the former after binding. We expect that such differences in the kinetic properties will affect the function of these PIPs *in cellulo* and *in vivo*. Moreover, experiments using disease models are currently underway.

**Figure 2:**
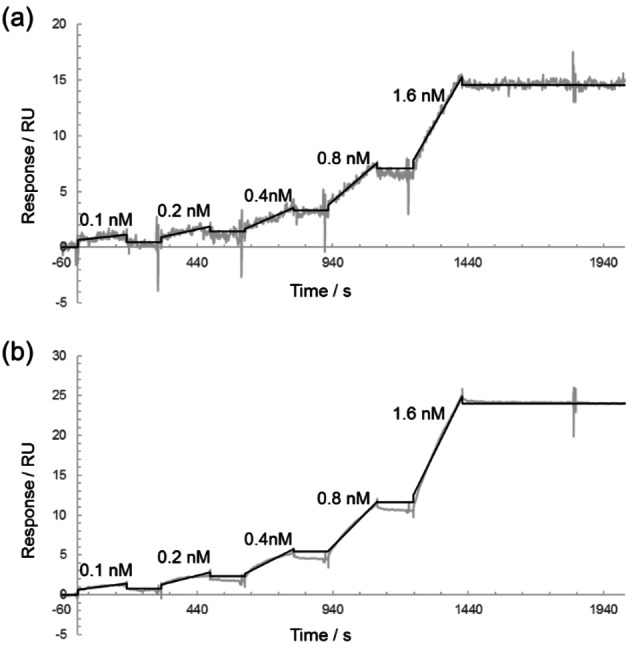
SPR sensorgrams using (a) cPIP **1** and (b) hPIP **2**. Gray and black curves represent the experimental data and fitting curves, respectively.

**Table 2:** Binding affinities of compounds **1** and **2**.

	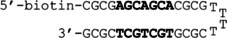		
PIP	*k*_a_/M^−1^s^−1^	*k*_d_/s^−1^	*K*_D_/M
cPIP **1**	4.1×10^4^	5.2×10^−7^	1.3×10^−11^
hPIP **2**	2.2×10^6^	2.7×10^−6^	1.2×10^−12^

All values are determined by fitting with a 1 : 1 binding model.

The findings of the *T*_m_ and SPR assays showed high binding affinity of cPIP **1** to its target DNA sequence. However, we had not considered its sequence specificity. To investigate the binding specificity of PIPs for a broad range of DNA sequences, the Bind‐n‐Seq method, which is a powerful tool that utilizes large‐scale parallel sequencing, can be used.[^17^, ^18^, ^19^, ^20^] We prepared a biotinylated cPIP (cPIP **3** in Figure 3a) and performed the Bind‐n‐Seq method using random DNA sequences as described previously.^[21]^

**Figure 3:**
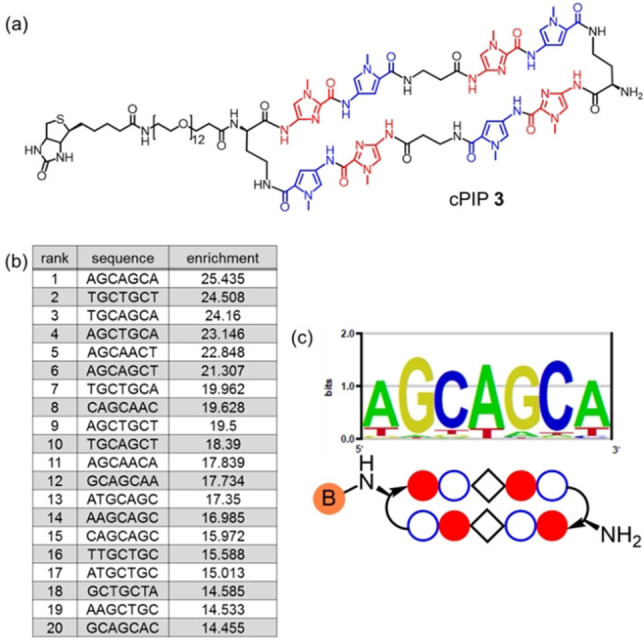
(a) The chemical structure of compound **3**. (b) Seven‐bp sequences in the order of enrichment values. (c) Motif analysis.

The enriched sequences are shown in Figure 3b in the order of enrichment values, and the binding motif result is presented in Figure 3c. The binding motif obtained using the Bind‐n‐Seq method matched almost perfectly with the target sequence of cPIP **3** (5′‐WGCWGCW‐3′). This finding suggests that the original PIP **1** recognizes its target sequence with high specificity.

In summary, we synthesized cPIP **1** and hPIP **2**, which target the CAG/CTG repeat sequences, and examined their DNA‐binding properties *in vitro*. The findings of the *T*_m_ assay indicated that cPIP **1** has a higher binding affinity than hPIP **2**. The SPR assays revealed that these PIPs possess different kinetic properties on binding to their target DNA sequence. In addition, the findings of the Bind‐n‐Seq method indicated that PIP **1** had high sequence specificity to the target DNA sequence. Taken together, these findings suggest that cPIP **1** can be used as a ligand to distinguish the CAG/CTG repeat sequences. Further experiments using cellular and animal disease models should be performed to develop the use of cPIP **1** as a therapeutic drug and molecular probe.

## Experimental Section

**General**: Reagents and solvents were purchased from standard suppliers and used without further purification. HPLC analysis was performed on a Jasco Engineering PU‐2089 plus series system using a COSMOSIL 150×4.6 mm 5 C_18_‐MS‐II Packed Column (Nacalai Tesque, Inc.) in 0.1 % TFA in water with acetonitrile as the eluent at a flow rate of 1.0 mL/min and a linear gradient elution of 0–100 % acetonitrile in 40 min with detection at 254 nm. Collected fractions were analyzed by MALDI‐TOF‐MS microflex‐KS II (Bruker). HPLC purification was carried out by Jasco engineering PU‐2080 or PU‐2089 plus series using a COSMOSIL 150×10 mm 5 C_18_‐MS‐II Packed Column (Nacalai Tesque, Inc.) in 0.1 % TFA in water with acetonitrile as the eluent at a flow rate of 3.8 mL/min and a linear gradient elution of +1 %/min acetonitrile for 20 min with detection at 254 nm. Master DMSO solutions of PIP were prepared based on the following formula.

ϵ=9900×(sumnumberofPyandIm)

Abs=ϵcl

ϵ, Abs, c, and l are molar extinction coefficients of PIPs in DMSO solution at around 302 nm, absorbance at 302 nm measured by Nanodrop 1000 spectrophotometer (Thermo Fisher Scientific Inc.), molar concentration, and the path length, respectively. ^1^H‐NMR spectra were measured on JEOL JNM ECA‐600 spectrometer (600 MHz for ^1^H), with chemical shifts reported in parts per million relative to residual solvent and coupling constants in hertz. The following abbreviations were applied to spin multiplicity: s (singlet), d (doublet), t (triplet), and m (multiplet).

**Fmoc solid‐phase synthesis of PIPs**: The solid‐phase synthesis of each PIP was performed on a PSSM‐8 system (Shimadzu) as described previously.^[8]^ The building blocks used in this study are FmocHN‐Py‐CO_2_H, FmocHN‐Im‐CO_2_H, FmocHN‐Py‐β‐CO_2_H, FmocHN‐Boc‐(*R*)‐α‐aminobutyric acid. Each of them was introduced sequentially to FmocHN‐Py‐trityl resin (for **1**) or FmocHN‐Py‐oxime resin (for **2**). The N‐terminus of hairpin PIP (**2**) was capped by acetyl group using 20 % Ac_2_O in DMF.

**cyclo‐(‐ImPyβImPy‐(*R*)^α−NH2^γ‐ImPyβImPy‐(*R*)^α−NH2^γ‐) (1)**: Using 86 mg (49 mg+37 mg) FmocHN‐Py‐trityl resin (0.335 mmol/g+0.137 mmol/g) and proper building blocks, H_2_N‐(*R*)^α−NHBoc^γ‐ImPyβImPy‐(*R*)^α−NHBoc^γ‐ImPyβImPy‐trityl resin was synthesized by the Fmoc solid‐phase synthesis. After H_2_N‐(*R*)^α−NHBoc^γ‐ImPyβImPy‐(*R*)^α−NHBoc^γ‐ImPyβImPy‐trityl resin was cleaved with 30 % hexafluoroisopropanol (HFIP) in dichloromethane (DCM) for 3 hours at room temperature, resin was removed by filtration and the filtrate was dropped into Et_2_O to obtain 26.1 mg brown powder of H_2_N‐(*R*)^α−NHBoc^γ‐ImPyβImPy‐(*R*)^α−NHBoc^γ‐ImPyβImPy‐CO_2_H (Analytical HPLC: t_R_=18.3 min. MALDI‐TOF MS: *m/z* calcd for C_68_H_89_N_26_O_17_^+^ [M+H]^+^ 1541.68, found; 1541.75). This crude sample was dissolved in DMF 16.9 mL (1 mM) for the next intramolecular cyclization step. After the addition of pentafluorophenyl diphenylphosphinate (FDPP, 3 equiv.) and *N,N*‐diisopropilethylamine (DIEA, 6 equiv.), the mixture was stirred for 24 hours at room temperature. Then the solvent was evaporated and dried *in vacuo*. The vacuum‐dried brown oil was dissolved in the minimum volume of MeOH/DCM 1 : 1 mixture dropped into Et_2_O. Then Et_2_O was removed *in vacuo* to obtain 23.9 mg brown powder of cyclo‐(‐ImPyβImPy‐(*R*)^α−NHBoc^γ‐ImPyβImPy‐(*R*)^α−NHBoc^γ‐). This crude sample was dissolved in trifluoroacetic acid (TFA)/DCM 2 : 5 mixture 700 μL and stirred for 30 minutes at room temperature to deprotect two Boc groups. After the reaction, the reaction mixture was dropped into Et_2_O and powdered. The powder was dried *in vacuo* and 23.5 mg brown powder of cyclo‐(‐ImPyβImPy‐(*R*)^α−NH2^γ‐ImPyβImPy‐(*R*)^α−NH2^γ‐) (**1**) was obtained. A part of this crude sample (7.9 mg) was dissolved in DMF and purified by HPLC. 1.2 mg of purified sample was obtained as off‐white powder (0.91 μmol, 13 % yield for 13 steps). Analytical HPLC: t_R_=13.5 min. MALDI‐TOF MS: *m/z* calcd for C_58_H_71_N_26_O_12_^+^ [M+H]^+^ 1323.57, found;1323.69. ^1^H‐NMR (600 MHz, DMSO‐d_6_): δ 11.02 (s, 2H), 10.25 (s, 2H), 9.98 (s, 2H), 9.97 (s, 2H), 8.31 (d, J=4.2 Hz, 6H), 8.20 (t, J=5.4 Hz, 2H), 8.04 (t, J=6.0 Hz, 2H), 7.50 (s, 2H), 7.46 (s, 2H), 7.30 (d, J=1.8 Hz, 2H), 7.24 (d, J=1.8 Hz, 2H), 7.00 (d, J=1.8 Hz, 2H), 6.82 (d, J=1.8 Hz, 2H), 4.05–4.00 (m, 2H), 3.95 (s, 6H), 3.94 (s, 6H), 3.81 (s, 12H), 3.28–3.23 (m; partially overlapped with H_2_O, 8H), 2.60–2.57 (m; partially overlapped with DMSO, 4H), 2.00–1.96 (m, 4H).

**AcImPyβImPy‐(*R*)^α−NH2^γ‐ImPyβImPyDp (2)**: Using 85 mg FmocHN‐Py‐oxime resin (0.328 mmol/g) and proper building blocks, AcImPyβImPy‐(*R*)^α−NHBoc^γ‐ImPyβImPy‐oxime resin was synthesized by the Fmoc solid‐phase synthesis. After AcImPyβImPy‐(*R*)^α−NHBoc^γ‐ImPyβImPy‐oxime resin was cleaved with *N,N*‐dimethyl‐1,3‐propanediamine (Dp) for 3 hours at 55 °C, resin was removed by filtration and the filtrate was dropped into Et_2_O to obtain 37.0 mg brown powder of AcImPyβImPy‐(*R*)^α−NHBoc^γ‐ImPyβImPyDp (Analytical HPLC: t_R_=18.2 min. MALDI‐TOF MS: *m/z* calcd for C_66_H_87_N_26_O_14_^+^ [M+H]^+^ 1467.68, found; 1467.66). This crude sample was dissolved in TFA/DCM 2 : 5 mixture 700 μL and stirred for 30 minutes at room temperature to deprotect a Boc group. After the reaction, the reaction mixture was dropped into Et_2_O and powdered. The powder was dried *in vacuo* and 46.4 mg brown powder of AcImPyβImPy‐(*R*)^α−NH2^γ‐ImPyβImPyDp) (**2**) was obtained. A part of this crude sample (10.7 mg) was dissolved in DMF and purified by HPLC. 4.4 mg of purified sample was obtained as off‐white powder (3.2 μmol, 50 % yield for 12 steps). Analytical HPLC: t_R_=14.4 min. MALDI‐TOF MS: *m/z* calcd for C_61_H_79_N_26_O_12_^+^ [M+H]^+^ 1367.63, found;1367.78.

**Cyclo‐(‐ImPyβImPy‐(*R*)^α−NH−PEG12−Biotin^γ‐ImPyβImPy‐(*R*)^α−NH2^γ‐) (3)**: The crude powder of compound (4.0 mg) **1** was mixed with DIEA (6 equiv.) and EZ‐Link^TM^ NHS‐PEG_12_‐Biotin (Thermo Fisher Scientific, 0.7 equiv.) in 50 μL of DMF. The mixture was stirred for 25 min at r.t. Evaporation of the solvent yielded a yellow oil, which was purified by HPLC to afford compound **3** as a light yellow powder (0.4 mg, 0.30 μmol, 5 % yield for 14 steps). HPLC and MALDI‐TOF mass spectroscopy were carried out to identify the synthetic compound. Analytical HPLC: t_R_=16.0 min. MALDI‐TOF MS: *m/z* calcd for C_95_H_138_N_29_O_27_S^+^ [M+H]^+^ 2149.0, found; 2149.13.

***T***_**m**_ **analysis**: Four DNA oligomers (5’‐CGAGCAGCACG‐3’/3’‐GCTCGTCGTGC‐5’, 5’‐(CAG)_10_‐3’ and 5’‐(CTG)_10_‐3’) used in this analysis were purchased from Sigma and two RNA oligomers (5’‐r(CAG)_10_‐3’ and 5’‐r(CTG)_10_‐3’) were purchased from Thermo Fisher. The analytical buffer for *T*_m_ analysis was an aqueous solution of 2.5 mM sodium chloride and 10 mM Tris‐HCl at pH 7.5 containing 0.375 % v/v DMSO. The concentration of dsDNA was 2.5 μM. The concentration of polyamides was 3.75 μM (1.5 equiv.). Before the analysis, the samples were annealed from 95 °C to 20 °C at a rate of 1.0 °C/min. Absorbance at 260 nm was recorded from 20 °C to 95 °C at a rate of 1.0 °C/min using a spectrophotometer V‐750 (JASCO) with a thermocontrolled PAC‐743R cell changer (JASCO) and a thermalcirculator CTU‐100 (JASCO). The *T*_m_ values shown in Table 1 and Figures S5–S7 are the average of all data (n≥2). The representative denaturing graphs of each compound are shown in Figures S5–S7.

**SPR assays**: SPR assays were performed on a Biacore T200 instrument (GE Healthcare) following the manufacturer's instruction. A biotinylated DNA oligomer (5’‐biotin‐CGCGAGCCAGCACGCGTTTTCGCGTGCTGCTCGCG‐3’) was purchased from Sigma and immobilized to the streptavidin‐functionalized SA sensor chip (GE Healthcare) to obtain the desired immobilized level (approximately 630 RU rise). SPR assays were carried out using HBS‐EP buffer (GE Healthcare, 10 mM HEPES pH 7.4, 150 mM NaCl, 3 mM EDTA, and 0.005 % Surfactant P20) with 0.1 % DMSO at 25 °C. A series of sample solutions with various concentrations were prepared in HBS‐EP buffer with 0.1 % DMSO. The contact time, dissociation time and flow rate were set for 180 s, 600 s, and 100 μL/min, respectively. To measure the rates of association (*k*_a_) and dissociation (*k*_d_) and dissociation constant (*K*_D_), data processing was performed by using the Biacore T200 Evaluation Software version 1.0. The sensorgrams were fitted by using a 1 : 1 binding model. All sensorgrams and all values are shown in Figures 2 and S9 and Table 2.

**Bind‐n‐Seq analysis**: Bind‐n‐Seq analysis was conducted with an Ion PI System (Thermo Fisher Scientific). Bind‐n‐Seq and subsequent analysis to evaluate small‐molecule binding affinity towards specific DNA sequences in a broad context sequence pool were modified for the Ion Proton sequencer (ThermoFisher Scientific). The scheme involves three major steps.

1. Synthesis of biotinylated PIPs and randomized oligonucleotides with high‐throughput sequencing platform‐specific adapters (Ion torrent PI; oligonucleotides consisting of Ion Torrent sequencing library adapter A1, Ion Express Barcode, 21‐mer randomized sequence, and another adapter P1 were purchased from Sigma‐Aldrich). Oligonucleotides (3 μM) were duplexed by primer extension with adapter‐specific primer 1 (9 μM) in 25 mL reactions containing GoTaq Green (Promega) PCR master mix (2×) with Mg^2+^ (2 mM). Reactions were performed at 95 °C (2 min), 63 °C (1 min), 72 °C (4 min), and then 4 °C in a thermocycler (BioRad). Biotin‐conjugated PIPs (100 nM) were allowed to equilibrate with duplex random oligonucleotides for 20 h followed by the addition of Dynabeads M‐280 Streptavidin (Thermo Fisher Scientific; beads prepared based on the previous report), then separation of bound and unbound sequences by affinity purification.
2. Polyamide‐enrichment recovered DNA was diluted (1 : 10) and amplified with sequencing library adapter‐specific primer for 15 cycles to obtain sufficient sequencing template. After purification, enriched libraries were subjected to quality and quantity checking with a DNA High sensitivity BioAnalyzer kit (Agilent Technologies). The qualified libraries were used for template preparation by using an Ion PI Hi‐Q OT2 200 kit in an Ion OneTouch 2 system (Thermo Fisher Scientific). The templates were then enriched by using an Ion OneTouch ES enrichment system. The enriched libraries were sequenced by single‐read sequencing (Ion Proton sequencer with Ion PI Hi‐Q sequencing 200 kit and Ion PI Chip v3; Thermo Fisher Scientific) by following the manufacturer's instructions.
3. The sequenced reads (A, C, T, and G) were then processed to obtain a valid constant region and unique random region and retained and split into separate files through a unique 10‐nt ion Xpress Barcode. To count the number of PIP enriched unique DNA sequences, a sliding window (length, 7) in MERMADE, and a new pipeline for Bindn‐Seq analysis (http://korflab.ucdavis.edu/Datasets/BindNSeq) were used. The motifs were processed by enoLOGOS (http://www.benoslab.pitt.edu/cgi‐bin/enologos/enologos.cgi).

## Conflict of interest

The authors declare no conflict of interest.

## Supporting information

As a service to our authors and readers, this journal provides supporting information supplied by the authors. Such materials are peer reviewed and may be re‐organized for online delivery, but are not copy‐edited or typeset. Technical support issues arising from supporting information (other than missing files) should be addressed to the authors.

Supporting Information
