# Supplementary material for: Strong and Specific Recognition of CAG/CTG Repeat DNA (5’‐dWGCWGCW‐3’) by a Cyclic Pyrrole‐Imidazole Polyamide
Source: Chembiochem. 2021 Nov 18;23(2):e202100533. doi: 10.1002/cbic.202100533 (PMC9298716; doi:10.1002/cbic.202100533)
Supplement: Supplementary file 1 — Supporting Information [file CBIC-23-0-s001.pdf]

ChemBioChem

Supporting Information

Strong and Specific Recognition of CAG/CTG Repeat DNA (5′-dWGCWGCW-3′) by a Cyclic Pyrrole-Imidazole Polyamide

Yuki Hirose, Tomo Ohno, Sefan Asamitsu, Kaori Hashiya, Toshikazu Bando,* and Hiroshi Sugiyama*

CONTENTS

S2-S3. HPLC profiles and mass spectra of compounds **1−3** (figure S1-S3)

S4. NMR spectrum of compound **1** (figure S4)

S4-S5. Representative denaturation profiles in the T_m assays (Figure S5-S7)

S5. Brief explanation of the principle of the SPR method (Figure S8)

S6. SPR sensorgrams of compound **1** (Figure S9)

1

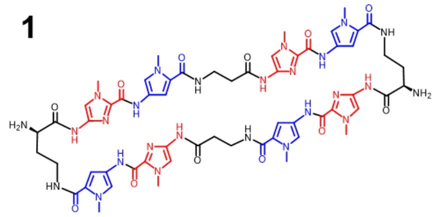

(a)

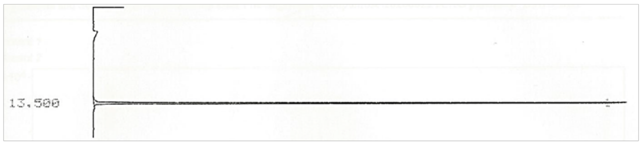

(b)

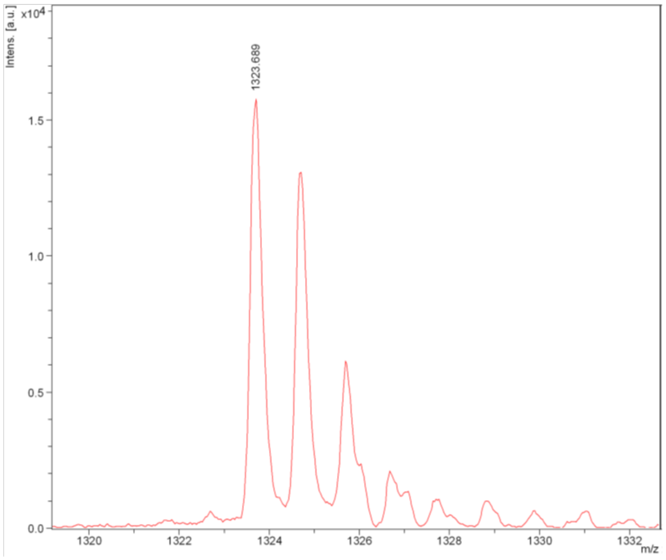

Figure S1. (a) HPLC spectrum of compound **1**. Conditions: 0.1% TFA containing 0−100% acetonitrile over a linear gradient for 40 min at a flow rate of 1.0 mL/min detected at 254 nm. The retention time was 13.500 min. (b) MALDI-TOF MS spectrum of compound **1**. *m/z* calcd for $C_{58}H_{71}N_{26}O_{12}^{+}$[M+H]$^{+}$1323.57, found; 1323.69.

2

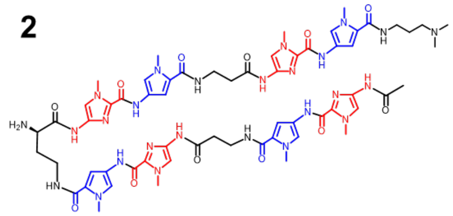

(a)

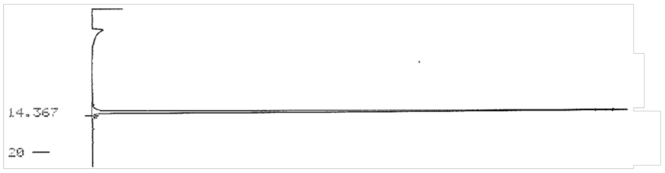

(b)

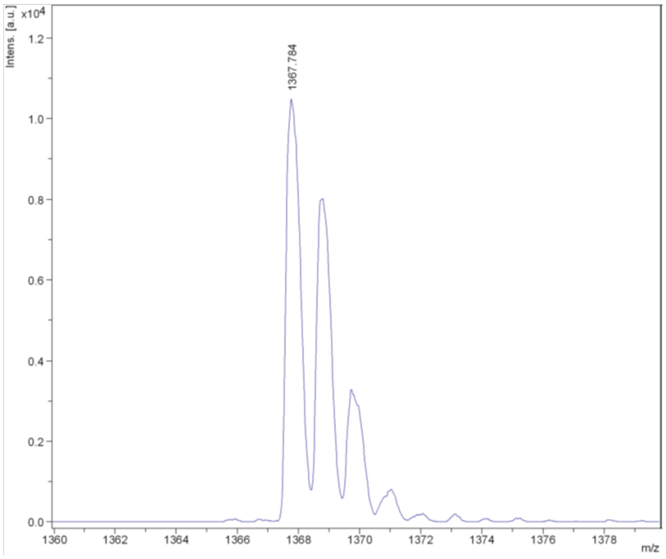

Figure S2. (a) HPLC spectrum of compound **2**. Conditions: 0.1% TFA containing 0−100% acetonitrile over a linear gradient for 40 min at a flow rate of 1.0 mL/min detected at 254 nm. The retention time was 14.367 min. (b) MALDI-TOF MS spectrum of compound **2**. *m/z* calcd for $C_{61}H_{79}N_{26}O_{12}^{+}$ [M+H]$^{+}$ 1367.63, found; 1367.78.

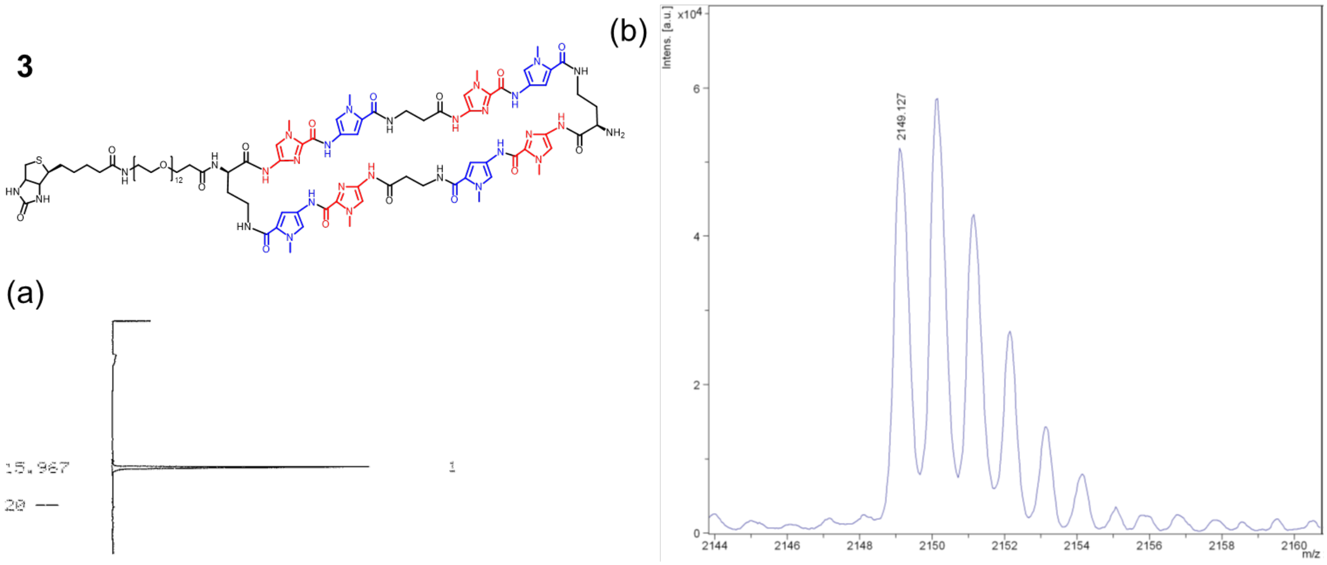

Figure S3. (a) HPLC spectrum of compound **3**. Conditions: 0.1% TFA containing 0−100% acetonitrile over a linear gradient for 40 min at a flow rate of 1.0 mL/min detected at 254 nm. The retention time was 15.967 min. (b) MALDI-TOF MS spectrum of compound **3**. *m/z* calcd for $C_{95}H_{138}N_{29}O_{27}S^{+}$ $[M+H]^{+}$ 2149.00, found; 2149.13.

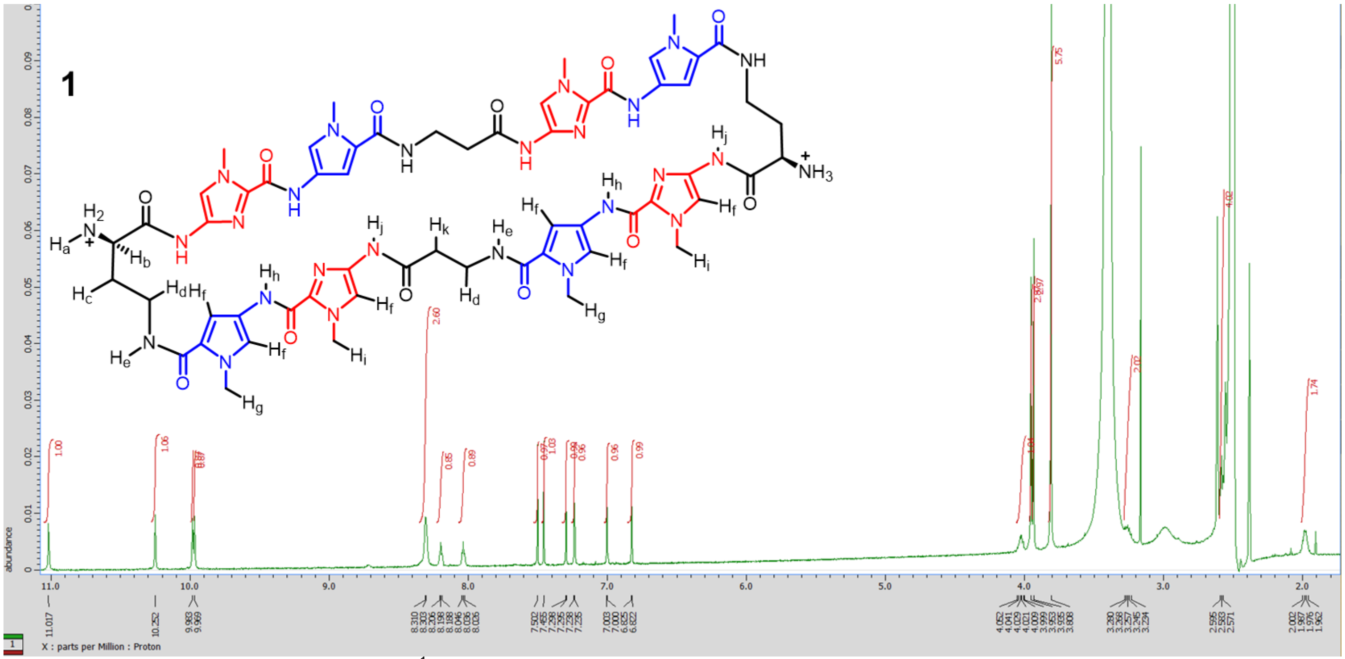

Figure S4. NMR spectrum of compound **1**. ^{1}H-NMR (600 MHz, DMSO-d_6) δ 11.02 (s, 2H, H_h), 10.25 (s, 2H, H_h), 9.98 (s, 2H, H_j), 9.97 (s, 2H, H_j), 8.31 (d, J = 4.2 Hz, 6H, H_a), 8.20 (t, J = 5.4 Hz, 2H, H_e), 8.04 (t, J = 6.0 Hz, 2H, H_e), 7.50 (s, 2H, H_f), 7.46 (s, 2H, H_f), 7.30 (d, J = 1.8 Hz, 2H, H_f), 7.24 (d, J = 1.8 Hz, 2H, H_f), 7.00 (d, J = 1.8 Hz, 2H, H_f), 6.82 (d, J = 1.8 Hz, 2H, H_f), 4.05-4.00 (m, 2H, H_b), 3.95 (s, 6H, H_g), 3.94 (s, 6H, H_g), 3.81 (s, 12H, H_i), 3.28-3.23 (m; partially overlapped with H_2O 8H, H_d), 2.60-2.57 (m; partially overlapped with DMSO, 4H, H_k), 2.00-1.96 (m, 4H, H_c).

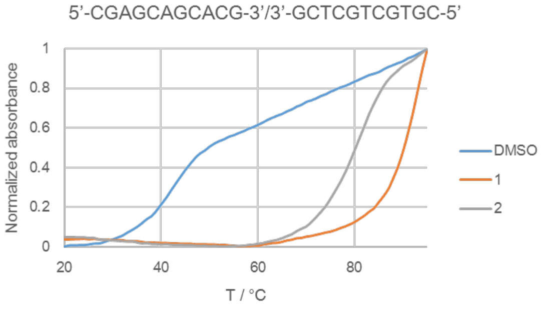

PIP	T_m / °C	ΔT_m / °C
-	41.9(± 0.5)	-
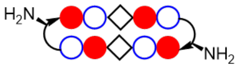 **1**	93.2 (± 0.2)	51.2 (± 0.6)
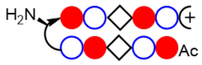 **2**	80.7 (± 0.4)	38.8 (± 0.7)

Figure S5. Representative denaturation profiles and calculated T_m and ΔT_m values of DNA (5’-CGAGCAGCACG-3’/3’-GCTCGTCGTGC-5’) with or without PIPs in the T_m assay.

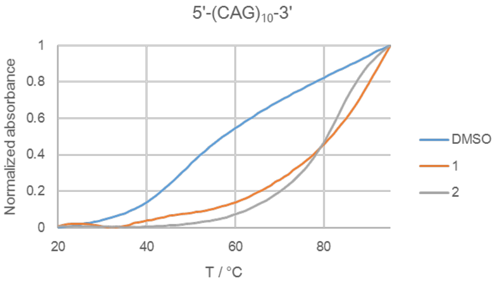

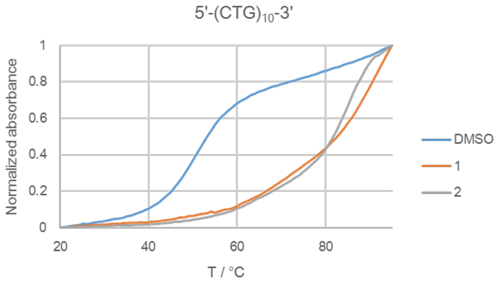

PIP	T_m / °C	ΔT_m / °C	T_m / °C	ΔT_m / °C
-	49.0 (± 0.7)	-	50.6 (± 0.3)	-
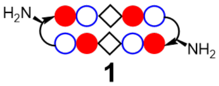 **1**	92.7 (± 0.5)	43.7 (± 0.8)	92.0 (± 0.6)	41.4 (± 0.7)
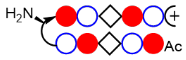 **2**	83.2 (± 0.6)	34.3 (± 0.9)	85.1 (± 0.4)	34.5 (± 0.5)

Figure S6. Representative denaturation profiles and calculated T_m and ΔT_m values of DNA (5’-$(CAG)_{10}$-3’ or 5’-$(CTG)_{10}$-3’) with or without PIPs in the T_m assay.

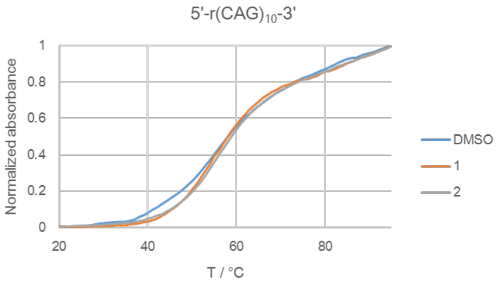

PIP	5'-r(CAG)10-3' T_m / °C	ΔT_m / °C	5'-r(CUG)10-3' T_m / °C	ΔT_m / °C
-	56.5 (± 0.9)	-	48.2 (± 0.3)	-
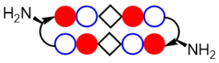 **1**	55.7 (± 0.6)	-0.8 (± 1.0)	48.6 (± 0.6)	0.3 (± 0.7)
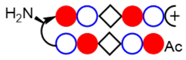 **2**	56.5 (± 0.6)	0.1 (± 1.0)	49.6 (± 0.6)	1.4 (± 0.7)

Figure S7. Representative denaturation profiles and calculated T_m and ΔT_m values of DNA (5'-r(CAG)$_{10}$-3' or 5'-r(CUG)$_{10}$-3') with or without PIPs in the T_m assay.

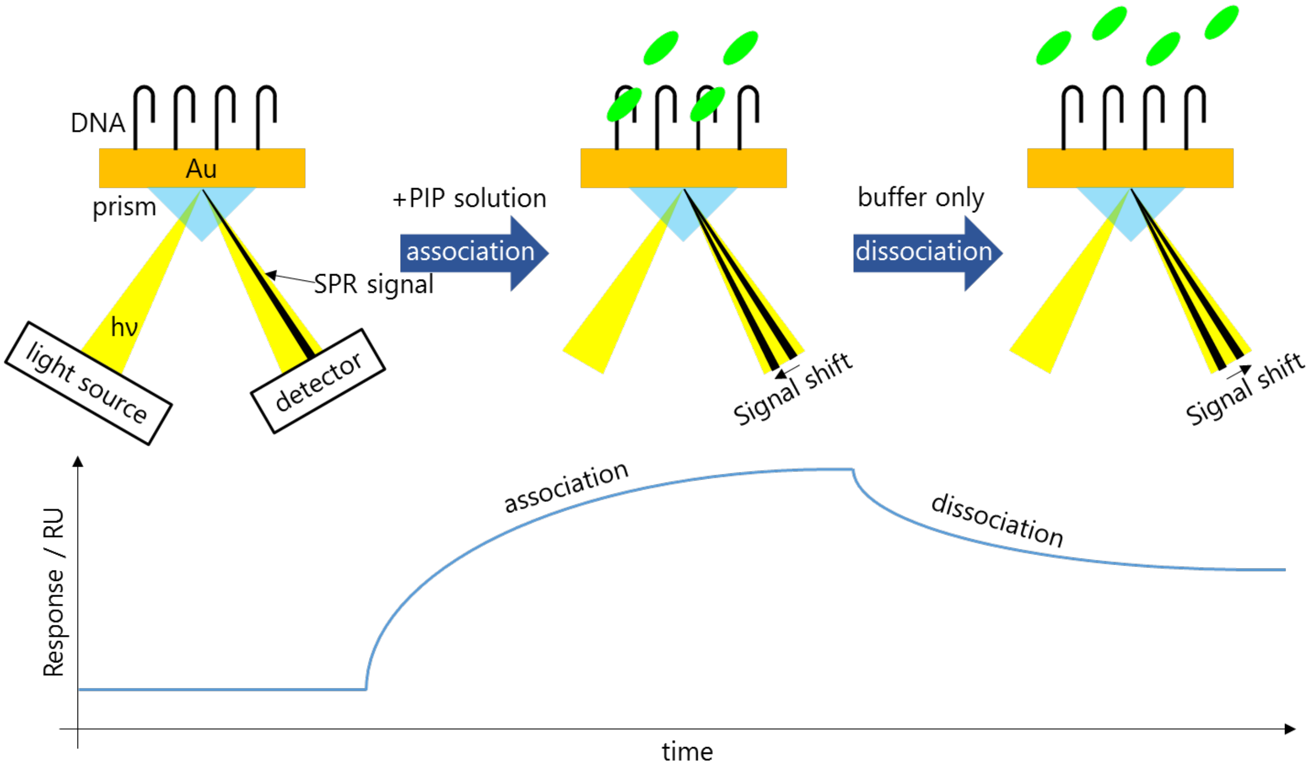

Figure S8. Brief explanation of the principle of the SPR method. 5'-biotin-labeled hairpin DNA was immobilized on a streptavidin-coated sensor chip, and the polyamide solution was added. The trace mass change associated with the binding and dissociation of PIP and DNA on the surface of the sensor chip is detected as an SPR signal and monitored as a graph (sensorgram). Parameters for molecular interactions (K_D, k_a, k_d, etc.) can be calculated from the sensorgram. RU (Resonance unit) is a unit that represents the degree of change in the SPR signal. 0.1° change in the SPR angle is defined as 1000 RU.

(a)

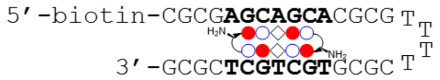

PIP	k_a / $M^{-1}s^{-1}$	k_d / s^{-1}	K_D / M	R_{max}	χ^2	U-value
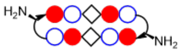 **1**	4.1×10^4	5.2×10^{-7}	1.3×10^{-11}	656.8	0.1	95

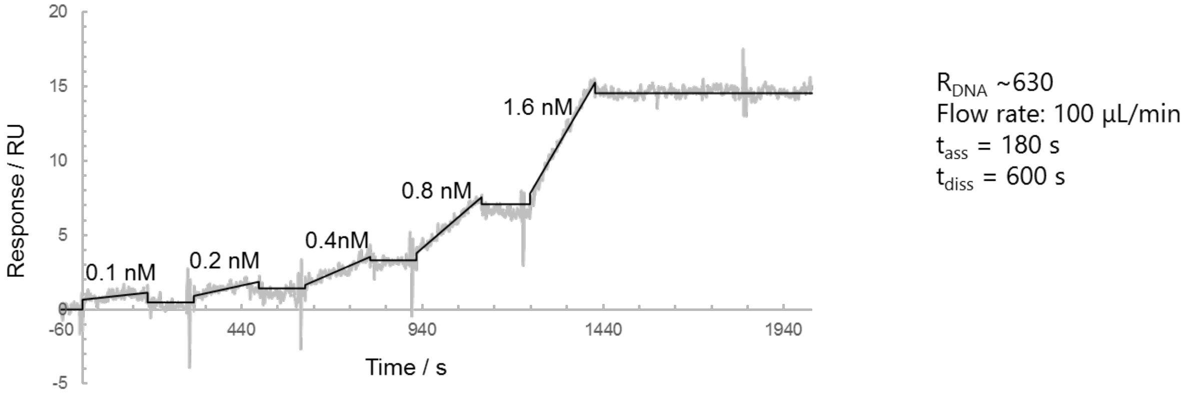

(b)

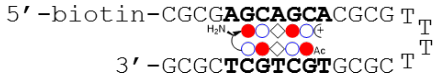

PIP	k_a / $M^{-1}s^{-1}$	k_d / s^{-1}	K_D / M	R_{max}	χ^2	U-value
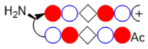 **2**	2.2×10^6	2.7×10^{-6}	1.2×10^{-12}	973.6	0.2	95

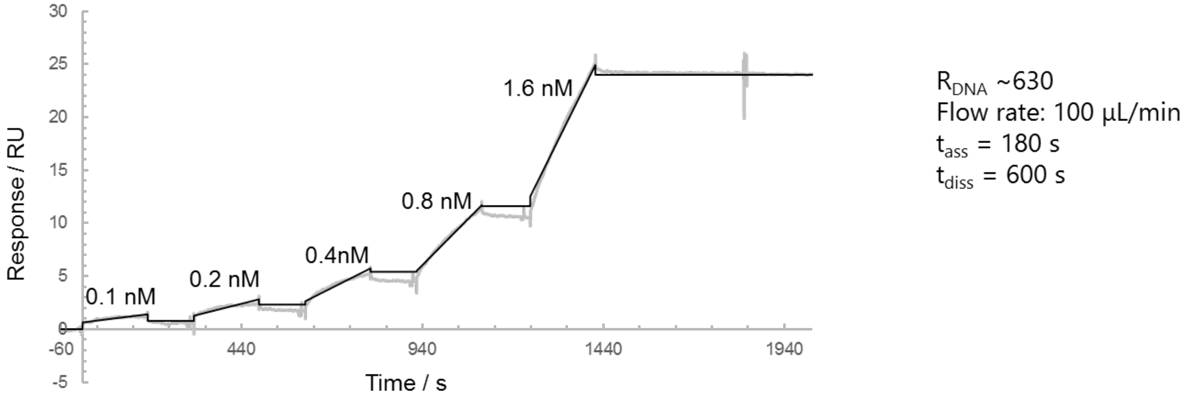

Figure S9. The SPR sensorgram and obtained values for (a) cPIP **1** and (b) hPIP **2**. R_{max} is the maximal binding amount when the analyte (PIP) occupies all of the immobilized ligand (DNA). The theoretical R_{max} can be calculated by using the following formula. R_{max} = Molecular weight of the analyte (Da) × Immobilized amount of the ligand (RU) × Number of binding site / Molecular weight of the ligand (Da).
